# Relationship between oxygen consumption and neuronal activity in a defined neural circuit

**DOI:** 10.1186/s12915-020-00811-6

**Published:** 2020-07-03

**Authors:** Suzan Özugur, Lars Kunz, Hans Straka

**Affiliations:** 1 Department Biology II, Ludwig-Maximilians-University Munich, Großhaderner Str. 2, 82152 Planegg, Germany; 2 Graduate School of Systemic Neurosciences, Ludwig-Maximilians-University Munich, Großhaderner Str. 2, 82152 Planegg, Germany

**Keywords:** *Xenopus laevis*, Oxidative phosphorylation, Ventricle, Extraocular motoneurons

## Abstract

**Background:**

Neuronal computations related to sensory and motor activity along with the maintenance of spike discharge, synaptic transmission, and associated housekeeping are energetically demanding. The most efficient metabolic process to provide large amounts of energy equivalents is oxidative phosphorylation and thus dependent on O_2_ consumption. Therefore, O_2_ levels in the brain are a critical parameter that influences neuronal function. Measurements of O_2_ consumption have been used to estimate the cost of neuronal activity; however, exploring these metabolic relationships in vivo and under defined experimental conditions has been limited by technical challenges.

**Results:**

We used isolated preparations of *Xenopus laevis* tadpoles to perform a quantitative analysis of O_2_ levels in the brain under in vivo-like conditions. We measured O_2_ concentrations in the hindbrain in relation to the spike discharge of the superior oblique eye muscle-innervating trochlear nerve as proxy for central nervous activity. In air-saturated bath Ringer solution, O_2_ levels in the fourth ventricle and adjacent, functionally intact hindbrain were close to zero. Inhibition of mitochondrial activity with potassium cyanide or fixation of the tissue with ethanol raised the ventricular O_2_ concentration to bath levels, indicating that the brain tissue consumed the available O_2_. Gradually increasing oxygenation of the Ringer solution caused a concurrent increase of ventricular O_2_ concentrations. Blocking spike discharge with the local anesthetics tricaine methanesulfonate diminished the O_2_ consumption by ~ 50%, illustrating the substantial O_2_ amount related to neuronal activity. In contrast, episodes of spontaneous trochlear nerve spike bursts were accompanied by transient increases of the O_2_ consumption with parameters that correlated with burst magnitude and duration.

**Conclusions:**

Controlled experimental manipulations of both the O_2_ level as well as the neuronal activity under in vivo-like conditions allowed to quantitatively relate spike discharge magnitudes in a particular neuronal circuitry with the O_2_ consumption in this area. Moreover, the possibility to distinctly manipulate various functional parameters will yield more insight in the coupling between metabolic and neuronal activity. Thus, apart from providing quantitative empiric evidence for the link between physiologically relevant spontaneous spike discharge in the brain and O_2_-dependent metabolism, isolated amphibian preparations are promising model systems to further dissociate the O_2_ dynamics in relation to neuronal computations.

## Background

The brain requires a disproportionately large amount of energy compared to its fraction of body mass [1]. This large amount of energy ensures maintenance of the functionality of the cellular components such as neurons and glial cells [2] and is indicated by the considerable O_2_ consumption by this organ [3]. The close correlation between energy demand and O_2_ supply derives from the fact that the generation of adenosine triphosphate (ATP) as most important energy equivalent occurs mainly via oxidative phosphorylation [4, 5]. This highly productive metabolic process takes place in mitochondria and requires considerable amounts of O_2_ [4]. In the brain, this aerobic generation of energy is assigned mostly, even though not exclusively to neurons. In contrast, glial cells largely depend on anaerobic mechanisms, producing lactate, which also fuels oxidative phosphorylation in neurons [6, 7]. Thus, O_2_ turnover has been used in the past in a number of experimental and theoretical studies to estimate the cost of neuronal activity from single-cell conductance to network computations (e.g., [8–10]). However, only few studies have provided reliable estimates of the O_2_ consumption under defined experimental conditions (e.g., neuronal activity patterns) because of the generally challenging technical requirement of O_2_ measurements and the difficult relation between consumption and spike firing [5, 8–12].

The ATP and O_2_ demand for specific neuronal functions such as action potential generation, maintenance of resting membrane potential, or integration of inhibitory and excitatory synaptic inputs [2, 13, 14] has often been determined only indirectly by measuring metabolites such as nicotinamide adenine dinucleotide (NADH) and flavin adenine dinucleotide (FAD) mostly in mammalian brain slice preparations or cell cultures [15–21]. A number of mainly theoretical considerations have demonstrated that particular neuronal processing features require a certain amount of energy in form of ATP and O_2_ [2, 22–25]. Some of these calculations have been confirmed by rather rare empiric experimental examples, mostly in slice preparations that were able to demonstrate a direct link between, e.g., neuronal spike discharge and O_2_ consumption [8–10, 12, 26], which are difficult to achieve in vivo in intact animals. This limitation is largely due to the massively invasive way of directly recording O_2_ concentrations under defined conditions using O_2_ electrodes along with the concurrent neuronal activity. This at least in part explains the current lack of larger sets of experimental data that allow a direct correlation of O_2_ levels and neuronal activity.

Isolated in vitro whole brains or entire heads of amphibians (frogs: [27]; Axolotl: [28]) or reptilian species (snakes: [29]; turtles: [30]) represent excellent alternatives to study more complex neuronal computations related to in vivo-like behavior. After isolation, these preparations are usually maintained in air-saturated bath solutions. Remarkably, such isolated preparations can be maintained functionally intact for several days and employed for extended periods of recordings of spike discharge (e.g., [29, 31]) or calcium transients (e.g., [28, 32]) during natural or electrical activation of sensory inputs. Despite the established use for systemic neuroscientific studies, only little is known about the requirements for the consumption of O_2_ by the tissue in such preparations or the relation between neuronal activity and the O_2_ level in the brain.

Here, we used isolated preparations of *Xenopus laevis* tadpoles to monitor the O_2_ level in the IV^th^ ventricle and adjacent hindbrain. In air-saturated bath Ringer solution, the O_2_ concentration in the ventricular compartment was negligible, but increased to bath O_2_ levels following metabolic inactivation of the brain with potassium cyanide (KCN) or after ethanol (EtOH) fixation. The suggestive consumption of the entirety of available O_2_ under control conditions was confirmed in experiments in which the spike discharge was blocked by the local anesthetic tricaine methanesulfonate (MS-222). A correlation between neuronal discharge and O_2_ level was further elucidated during spontaneous spike bursts in a specific motor nerve, indicating causality between discharge magnitude and O_2_ consumption. Preliminary data were previously published in abstract form [33].

## Results

### Oxygen concentration profile

**Fig. 1.**
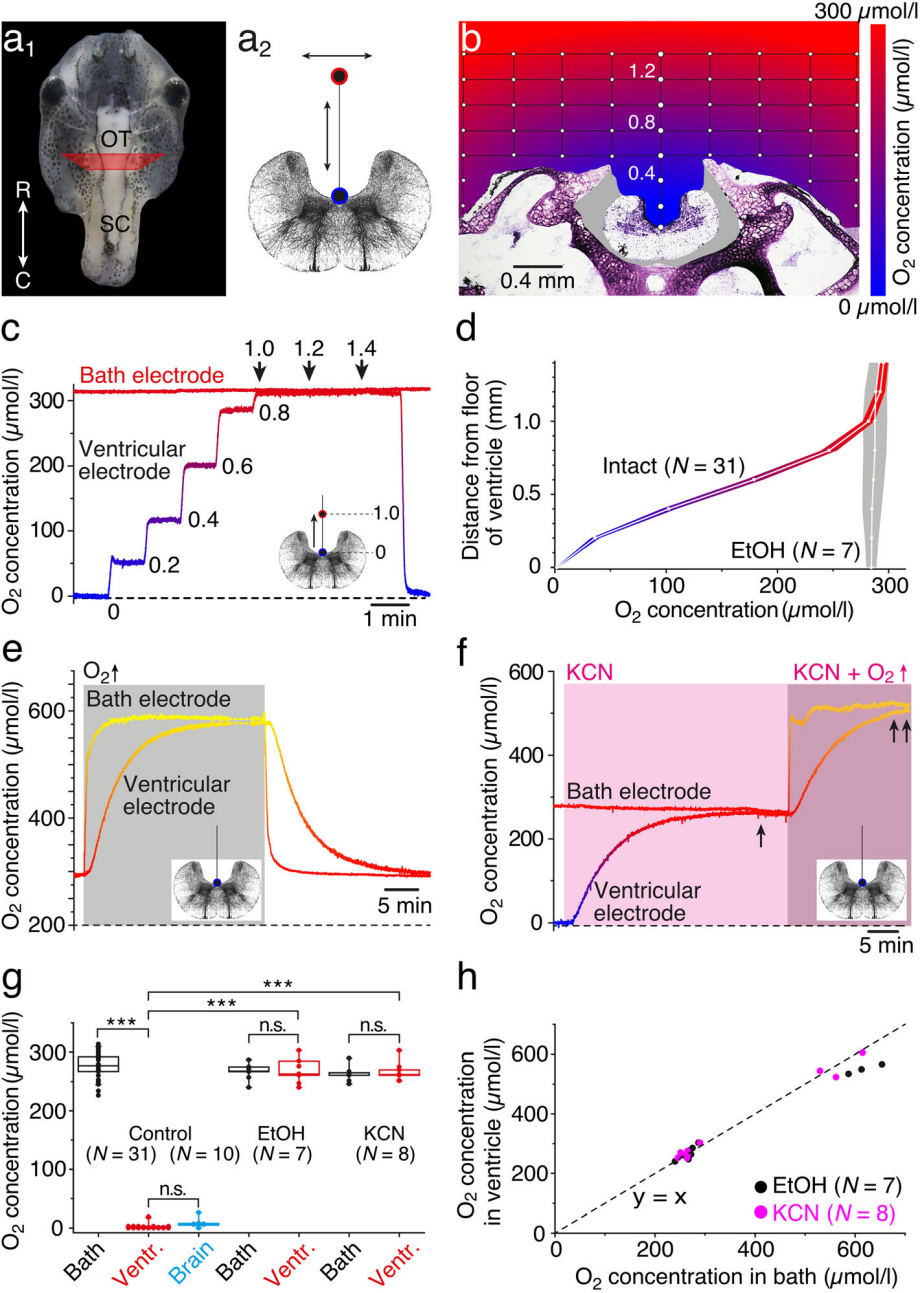
Measurements of O_2_ levels in isolated preparations. **a**, **b** Photomicrograph, depicting an isolated head of a stage 53 tadpole (**a**_**1**_), a schematic transverse section of the hindbrain (**a**_**2**_), and a cross-sectioned head (**b**) at a rostro-caudal level indicated by the trapezoid in **a**_**1**_; red and blue circles and arrows in **a**_**2**_ indicate movements of the O_2_ electrode within the grid (white dots in **b**), used to construct the O_2_ profile (**b**). **c** Dual recordings of O_2_ concentrations in the bath and above the floor of the IV^th^ ventricle in steps of 0.2 mm. **d** O_2_-concentration profile (mean ± SEM) of a midline depth track above the IV^th^ ventricle (**a**_**2**_) in control (intact; color-coded) and metabolically inactive (EtOH-fixated; gray) preparations. **e**, **f** Recording of the ventricular O_2_ concentration in an EtOH-fixated preparation (**e**) during temporary increase of the bath O_2_ level to 650 μmol/l (gray area), and of an intact preparation (**f**) after bath-application of KCN (light pink area; 500 μmol/l); note that KCN causes an adjustment of the ventricular O_2_ level to the bath O_2_ level (single arrow in **f**) that remains matched (double arrow in **f**) when the bath O_2_ level is further increased (dark pink area). **g** Boxplot, depicting O_2_ concentrations in air-saturated bath solution (black), at the ventricular floor (red), and within the hindbrain (blue) in controls, in EtOH-fixated and KCN-treated preparations; note that EtOH-fixation and bath-application of KCN cause a significant increase of ventricular O_2_ concentrations to bath Ringer levels (***p < 0.0001; Mann-Whitney *U* test). **h** Scatter plot depicting coinciding ventricular and bath O_2_ levels in metabolically inactive (black dots, EtOH-fixated) or oxidative phosphorylation-impaired (pink dots, KCN) preparations. O_2_ levels in **b**–**f** are color-coded from blue (0 μmol/l) to red (300 μmol/l) to yellow (600 μmol/l); transverse hindbrain schemes indicate motion (**c**) or position (**e**, **f**) of the O_2_ electrode. OT, optic tectum; R, rostral; C, caudal; SC, spinal cord; *N*, number of preparations

A potentially differential O_2_ distribution and gradient within and around the isolated preparation was tested by constructing a depth profile of the O_2_ concentration (Fig. 1a_2_, b–d). Following placement of the isolated preparation in the center of the recording chamber, one O_2_ electrode was positioned at a distance of 5 mm lateral to the preparation in a depth below the Ringer surface that matched the floor of the IV^th^ ventricle. Under control conditions with air-saturated Ringer solution (292 ± 4 μmol/l, mean ± SEM; *N* = 31 preparations), the second electrode was used to establish a step-wise dorso-ventral and medio-lateral profile of the O_2_ concentration within and around the tissue of the isolated preparation at the level of the hindbrain (see the “Methods” section). Measurements of the O_2_ level within the recording grid (white dots in Fig. 1b) and construction of a concentration map by extra- and interpolation revealed a differential distribution of the O_2_ levels around the tissue (color-coded map in Fig. 1b). While most of the Ringer solution above the preparation remained air-saturated (red in Fig. 1b), the region in proximity of the tissue (< 1.0 mm) became gradually more O_2_-depleted (violet in Fig. 1b), while the interior of the IV^th^ ventricle was entirely devoid of O_2_ (blue in Fig. 1b). Stepwise measurements along a depth track in the midline of the brain (in steps of 200 μm) in a typical example (Fig. 1c) illustrated the gradual reduction in O_2_ concentration (blue color) as the electrode approached the floor of the IV^th^ ventricle (ventricular electrode), while the Ringer in the recording chamber at a distance of 5 mm from the isolated preparation (bath electrode) remained at the same air-saturated level. This systematic difference in the O_2_ concentration between bath solution and ventricle was significant and observed in all preparations (control in Fig. 1g). Repetitive measurements along the depth track above the floor of the IV^th^ ventricle in a number of preparations (*N* = 31) confirmed the reproducibility of the gradient as indicated by the small variability (intact in Fig. 1d).

In order to demonstrate that metabolic activity in the hindbrain causes O_2_ depletion in the adjacent IV^th^ ventricle, some isolated preparations were fixated in 70% ethanol overnight (*N* = 7 preparations) and reused for O_2_ measurements. As the electrode along the depth track (gray area in Fig. 1d) approached the ventricular floor in these fixated preparations, the O_2_ concentration remained at the same level as the air-saturated Ringer solution in the bath (EtOH in Fig. 1d, g). Moreover, an increase of the O_2_ concentration in the bath (gray area) in these metabolically inactive preparations to ~ 600 μmol/l over a period of ~ 5 min as illustrated by the typical example in Fig. 1e (bath electrode) caused a delayed, but similar increase of the ventricular O_2_ concentration. The time to reach 95% of the steady-state level was ~ 15 min (14.8 ± 0.3 min; mean ± SEM; *N* = 7 preparations) and thus slower than the augmentation of the O_2_ level in the bath, due to the O_2_ diffusion into the ventricle. A comparable, matching dynamic of the O_2_ levels in the bath and the ventricle was observed during the return of the O_2_ concentration to air-saturated bath Ringer levels (right side in Fig. 1e). These findings suggest that (1) the shape of the IV^th^ ventricle generally poses no physical barrier for an increase/decrease of the O_2_ concentration when the bath O_2_ level is altered and that (2) the anoxic condition inside the ventricular compartment in intact preparations likely derives from the O_2_ consumption by the metabolically active tissue in the vicinity. In fact, measurements of the O_2_ concentration inside the hindbrain in functionally intact preparations exhibited similarly low levels as observed at the ventricular floor (compare ventr. and brain in Fig. 1g).

A causal relationship between the O_2_ consumption by the brain and the depleted ventricular O_2_ concentrations was further confirmed after impairing mitochondrial activity by bath-application of potassium cyanide (KCN; *N* = 8 preparations), which blocks cytochrome oxidase (complex IV) activity as the dominating O_2_-consuming process [34]. Bath application of 500 μmol/l KCN (light pink area in Fig. 1f) at air-saturated O_2_ concentrations of the bath Ringer solution (~ 290 μmol/l; bath electrode in Fig. 1f) gradually increased the O_2_-depleted ventricular level to a concentration that matched bath levels (ventricular electrode in Fig. 1f), compatible with the consequences of a complete block of the major O_2_-consuming metabolic processes. Further increase of the O_2_ level in the bath Ringer to > 500 μmol/l in the continuous presence of KCN (dark pink area in Fig. 1f) caused a delayed but matching augmentation of ventricular and bath O_2_ concentrations. Thus, in the absence of metabolic activity in the brain, either by fixation of the tissue with EtOH (Fig. 1e) or by bath-application of KCN (Fig. 1f), the normally O_2_-depleted ventricular compartment gradually reaches and maintains the same O_2_ level as the bath solution (see EtOH and KCN in Fig. 1g, h). Therefore, the negligible O_2_ concentration in metabolically unimpaired preparations likely reflects the O_2_ consumption by the brain, which directly relates to the anoxic level in the ventricle (compare ventr. and brain in Fig. 1g). Thus, the relatively hypoxic state of the ventricle is likely no pathological condition but rather an expression of the capacity of brain tissue to utilize efficiently all available O_2_. Accordingly, O_2_ meaurements in the IV^th^ ventricle represent an excellent proxy to monitor changes in O_2_ consumption by the brain with the additional benefit that the neuronal tissue remains undamaged since the tip of the O_2_ electrode is positioned in the ventricular compartment.

### Consequences of alterations in bath Ringer oxygenation

**Fig. 2.**
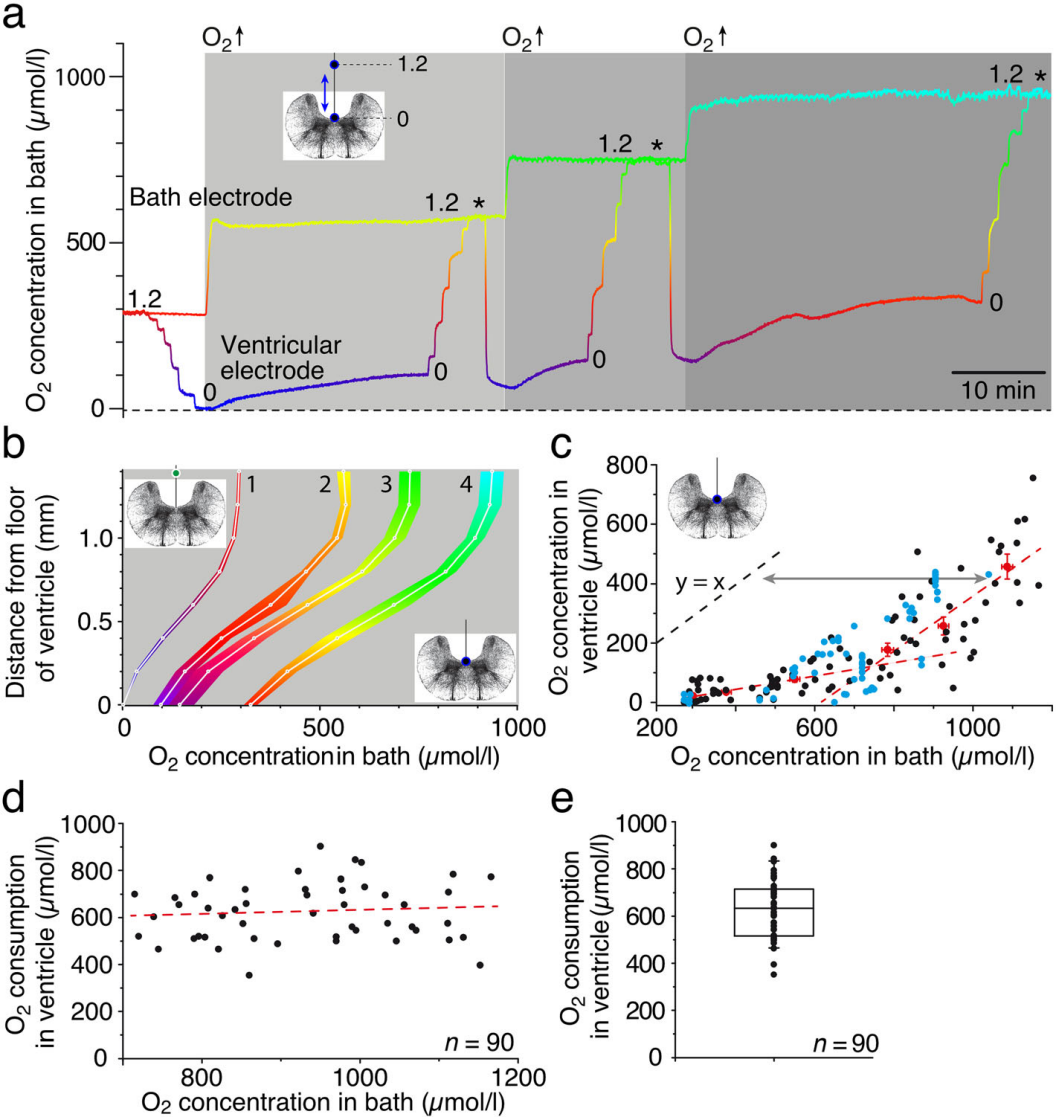
Influence of bath O_2_ concentrations on ventricular O_2_ levels. **a** Recording of O_2_ levels in the bath and ventricle following stepwise increase of the bath O_2_ concentration from ~ 290 μmol/l (air-saturated) to ~ 550 μmol/l (light gray area), ~ 750 μmol/l (gray area), and ~ 950 μmol/l (dark gray area); the O_2_-electrode was initially advanced to the ventricular floor in 0.2 mm steps (left in **a**) and transiently repositioned to 1.2 mm above the floor (*) prior to each increase of the bath O_2_ concentration. **b** O_2_ concentration profiles (mean ± SEM) of ventricular depth tracks (see insets) in air-saturated (~ 290 μmol/l) bath solution (1; *N* = 31) and after the increase of the bath O_2_ concentration to ~ 550 μmol/l (2; *N* = 6), ~ 750 μmol/l (3; *N* = 6), and ~ 950 μmol/l (4; *N* = 6). **c** Scatter plot, depicting the dependency of the ventricular O_2_ concentration (black and red dots; *n* = 97 from 24 preparations) and adjacent hindbrain (blue dots; *n* = 69 from 11 preparations) from bath O_2_ levels; red dots represent the mean ± SEM of the ventricular O_2_ level at distinct bath O_2_ concentrations and the red dashed lines linear regressions through the lower (*r*^2^ = 0.98) and higher (*r*^2^ = 0.96) range of mean ventricular concentrations, respectively. **d**, **e** Scatter plot (**d**) and boxplot (**e**) depicting ventricular O_2_ consumption as function of the bath O_2_ level for concentrations > 700 μmol/l (*n* = 90 from 13 preparations) with a mean ± SEM of 626 ± 13 μmol/l (**e**); the slope of the regression line in **d** (*r*^2^ = 0.007) is not significantly different from zero (*p* = 0.42). O_2_ levels in **a** and **b** are color-coded from blue (0 μmol/l) to red (300 μmol/l) to yellow (600 μmol/l) to green (750 μmol/l) to cyan (900 μmol/l); transverse hindbrain schemes indicate motion (**a**) or position (**b**, **c**) of the O_2_ electrode. *N*, number of preparations; *n*, number of measurements

The almost anoxic condition of the brain tissue, reflected by the level in the IV^th^ ventricle, prompted us to evaluate the consequences of elevated O_2_ concentrations in the bath solution on the O_2_ level in the tissue. In isolated preparations, under control condition with air-saturated bath Ringer solution (~ 290 μmol/l; bath electrode, left in Fig. 2a), the O_2_ electrode monitored the typical anoxic level as the electrode approached the floor of the IV^th^ ventricle (left in Fig. 2a). During stepwise increases of the O_2_ level in the bath solution (gray areas in Fig. 2a) to ~ 550 μmol/l (light gray area), to ~ 750 μmol/l (gray area) and to ~ 950 μmol/l (dark gray area), the ventricular O_2_ level of the typical example, shown in Fig. 2a, increased gradually to ~ 100 μmol/l, to ~ 150 μmol/l, and to ~ 350 μmol/l, respectively (see O_2_ values at 1.2 = bath level and 0 = ventricular floor in Fig. 2a). Confirmation of the validity of higher ventricular O_2_ measurements when increasing the O_2_ level in the bath Ringer was provided by a temporally brief retrieval of the electrode to 1.2 mm above the floor of the IV^th^ ventricle (* in Fig. 2a), a position where the O_2_ concentration reflects the bath solution.

Plots of dorso-ventral depth tracks along the midline of the hindbrain (schemes in Fig. 2b) with varying starting O_2_ levels of the bath Ringer, i.e., 290 μmol/l (air-saturated), 550 μmol/l, 750 μmol/l, and 950 μmol/l (see 1–4 in Fig. 2b) generally revealed very similar shapes of the gradients when the O_2_ electrode approached the ventricular floor. However, at variance with the control condition where the ventricular floor was found to be anoxic (1 in Fig. 2b; *N* = 31 preparations), elevated bath Ringer O_2_ levels were accompanied by gradually higher O_2_ concentrations at the ventricular floor (2–4 in Fig. 2b; *N* = 6 preparations, each). Interestingly, however, increases of the O_2_ concentration in the bath Ringer from ~ 290 μmol/l to ~ 750 μmol/l caused only a small augmentation by ~ 150 μmol/l at the ventricular surface (left red dashed line in Fig. 2c), whereas further increases of the bath O_2_ level to ~ 1200 μmol/l proportionally augmented the ventricular O_2_ level (right red dashed line in Fig. 2c). The small increase of ventricular O_2_ levels with bath levels up to ~ 750 μmol/l (*y* = 0.25x) suggests that the brain consumes most of the available O_2_. The slope close to unity (*y* = 0.93x) for bath Ringer O_2_ levels above ~ 750 μmol/l suggests that additionally provided O_2_ through the bath Ringer remains unused by the brain tissue and thus appears as corresponding increase of the ventricular O_2_ level (Fig. 2c). Calculating and plotting of the ventricular O_2_ consumption as difference between bath and actual ventricular O_2_ level (Fig. 2d; *n* = 90 measurements from 13 preparations) for bath O_2_ concentrations > 700 μmol/l, indicated that the consumed O_2_, although variable between preparations, is rather constant above this level as indicated by the linear regression with a slope that is not significantly different from zero (red dashed line in Fig. 2d; *p* = 0.42). Accordingly, the ventricular O_2_ consumption at bath levels between 700 and 1200 μmol/l was in the range of ~ 600 μmol/l (Fig. 2e). Importantly, O_2_ measurements within the adjacent hindbrain (blue circles in Fig. 2c) exhibited a very similar dependency from the bath Ringer O_2_ level, as the ventricle (black circles in Fig. 2c), indicating again that measurements in the latter compartment represent an excellent, minimal-damaging proxy for the O_2_ level of the adjacent central nervous tissue independent of the oxygenation level of the bath.

### Correlation between ventricular O_2_ levels and neuronal activity

**Fig. 3.**
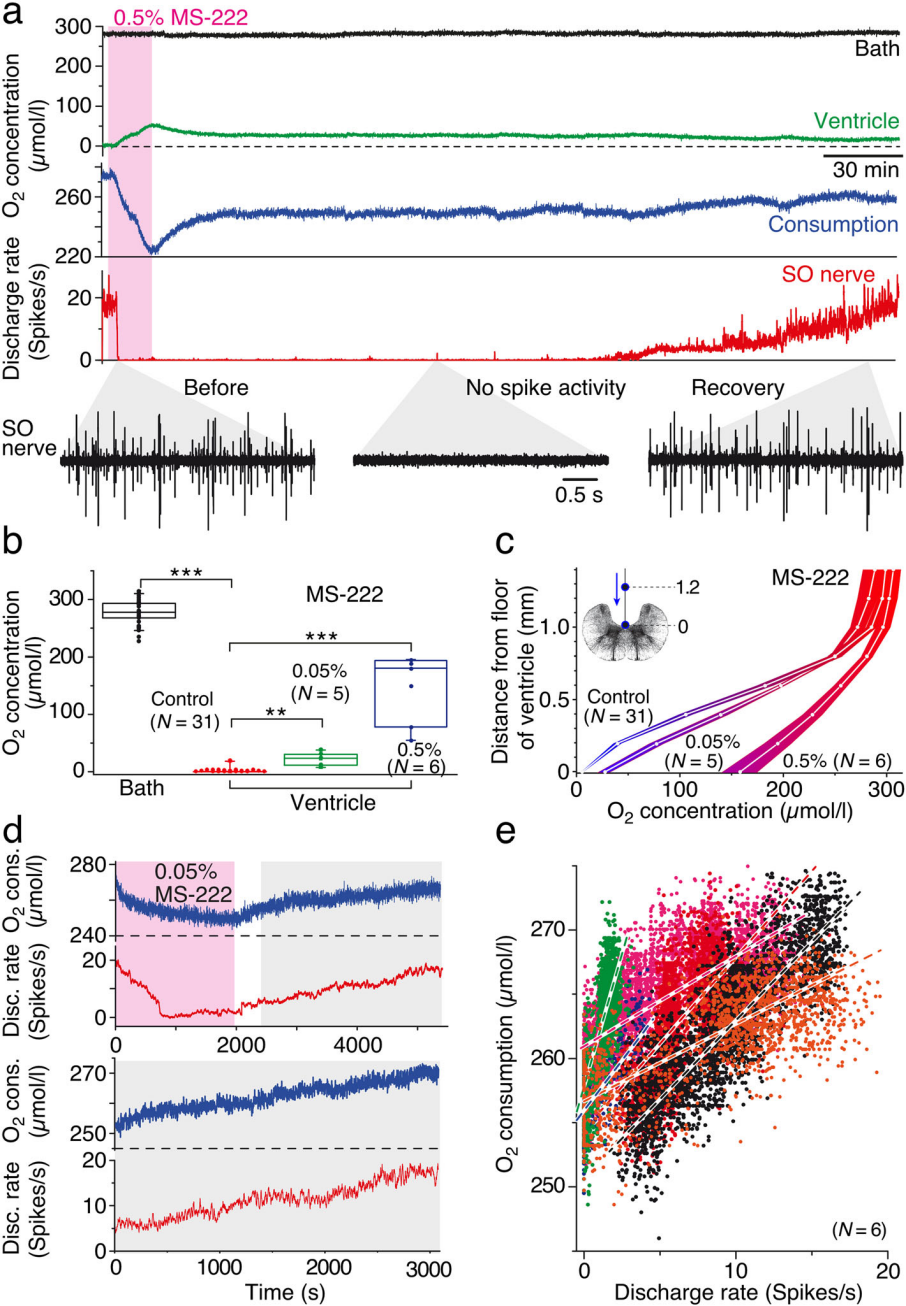
Correlation between neuronal discharge and ventricular O_2_ concentration. **a** Recording of O_2_ levels in the bath (top black trace) and ventricle (green trace) along with the resting rate (red trace) of superior oblique nerve spike activity illustrated at extended time scales (black traces) for selected periods before (before), after spiking has been blocked by bath-applied (0.5%; pink area) MS-222 (no spike activity), and during recovery (recovery); the O_2_ consumption (blue trace) was calculated as the difference of concurrent bath and ventricular O_2_ concentrations. **b** Boxplot, depicting O_2_ concentrations in the bath (black) and ventricle in controls (red; *N* = 31) and after bath-application of 0.05% (green; *N* = 5) and 0.5% MS-222 (blue; *N* = 6) indicating significantly increased ventricular O_2_ levels when the spike activity was blocked (**p < 0.001; ***p < 0.0001; Mann-Whitney *U* test. **c** O_2_ concentration profile (mean ± SEM) of a midline depth track above the floor of the IV^th^ ventricle in controls and after bath-application of 0.05% and 0.5% MS-222. **d** O_2_ consumption (upper blue trace), calculated from O_2_ concentrations of the bath Ringer and ventricle and corresponding superior oblique nerve discharge rate (upper red trace) during and after bath application of 0.05% MS-222 (pink area); lower traces illustrate the O_2_ consumption (blue) and resting rate (red) during the recovery from the drug effect at an extended time and amplitude scale (gray area from above). **e** Scatter plot, depicting the dependency of calculated O_2_ consumption on the resting rate of the superior oblique nerve during recovery from a 0.5% MS-222-induced block of spike activity (*N* = 6); data from individual preparations are color-coded; each linear regression line has a slope that is significantly different from zero (*p* < 0.0001); green *(r*^2^ = 0.52), blue (*r*^2^ = 0.42), red (*r*^2^ = 0.40), black (*r*^2^ = 0.71), pink (*r*^2^ = 0.55), and orange (*r*^2^ = 0.69). *N*, number of preparations

The major O_2_-consuming element in the brain are neurons, where oxidative phosphorylation is used to provide energy equivalents such as ATP to ensure the maintenance of the resting potential as well as the generation of inhibitory and excitatory responses including action potentials [5]. In fact, spike discharge and associated cellular homeostasis of the underlying conductances require the availability of considerable amounts of O_2_-dependent energy equivalents. This allows establishing a correlation between neuronal activity and O_2_ consumption. In fish and amphibians, MS-222, a tricaine derivate, is a potent blocker of action potentials through a prevention of activating the fast sodium conductance [35] and as such regularly used for the anesthesia of aquatic anamniotes [36]. To estimate the amount of O_2_ consumption in relation to neuronal activity in isolated *Xenopus* tadpole preparations, we employed extracellular recordings of multi-unit spike discharge of the superior oblique eye muscle-innervating motor nerve [37]. The superior oblique nerve resting discharge (lower black trace “before” in Fig. 3a) largely depends on inner ear afferent discharge and the corresponding spike activity of central vestibular neurons that tonically activate superior oblique motoneurons [38]. Both neuronal populations are located in the rostral hindbrain [39], in close proximity to the location of the ventricular O_2_ electrode. Accordingly, the extracellularly recorded multi-unit spike discharge of the superior oblique nerve provides a suitable correlate or proxy for central neuronal activity in a brain region adjacent to the IV^th^ ventricle.

Bath application of 0.5% MS-222 for ~ 15 min (pink area in Fig. 3a) reliably abolished the spontaneous discharge of the superior oblique nerve (black trace ‘No spike discharge’ and red trace in Fig. 3a). This effect was robust and required after the beginning of the washout ~ 3 h until the resting discharge was completely reestablished (black trace “recovery” and red trace in Fig. 3a). During MS-222 application, the typical anoxic condition at the ventricular floor transiently increased (green trace in Fig. 3a) in parallel with the cessation of the superior oblique nerve resting discharge (red trace in Fig. 3a). In the presence of a stable bath Ringer O_2_ level (upper black trace in Fig. 3a), this suggests that the O_2_ consumption of the neuronal tissue in the vicinity of the O_2_ electrode temporarily decreased (blue trace in Fig. 3a) in agreement with the MS-222-provoked blockage of neuronal activity. The augmentation of the ventricular O_2_ level following bath-application of 0.5% MS-222 was observed in all experiments (*N* = 6 preparations), reaching an average ventricular O_2_ level of ~ 150 μmol/l (blue box in Fig. 3b) that significantly differed from the low O_2_ level prior to MS-222 administration (*p* < 0.0001; Mann-Whitney *U* test). In contrast to the profound and relatively long-lasting effect of 0.5% MS-222, bath-application of a tenfold smaller concentration of MS-222 (0.05%) also provoked a smaller (~ 20 μmol/l), yet still significant increase of ventricular O_2_ levels (*p* < 0.0001; Mann-Whitney *U* test; *N* = 5 preparations; green box in Fig. 3b), a more transient block of the superior oblique motor nerve activity and a faster recovery to the anoxic control levels (not shown). The consistency of the dose-dependent drug effect across experiments was confirmed by construction of the O_2_-depth profile in the presence of 0.05% and 0.5% MS-222, respectively, illustrating the differential alteration of the gradient as the O_2_ electrode approached the ventricular floor (Fig. 3c). Thus, MS-222-provoked blockage of neuronal activity caused a reduced O_2_ consumption of the central nervous tissue confirming that action potential generation and associated maintenance of ion homeostasis is a dominant O_2_-consuming process in the brain, which is further elaborated in the discussion. The absence of firing rate recovery during the initial washout phase with a relatively early increase in O_2_ consumption might be related to the fact that MS-222 also blocks tetrodotoxin-insensitive Nav-type sodium channels, even though at lower affinity [40]. These ion channels are not involved in action potential generation but contribute to the energy-consuming maintenance of the resting membrane potential.

A correlation between neuronal activity and O_2_ consumption was obtained during the final recovery phase of spike firing after bath-applied MS-222 had temporarily extinguished the resting discharge of the superior oblique nerve (Fig. 3d). The close correspondence of resting discharge rate (red trace in Fig. 3d) and concurrent ventricular O_2_ consumption (blue trace in Fig. 3d) in all experiments (*N* = 6 preparations) suggests that the increase in O_2_ consumption during the final washout phase of MS-222 is causally related to the parallel reacquisition of the spontaneous discharge. In fact, plotting the gradually increasing O_2_ consumption during this period with respect to the superior oblique nerve firing rate reveals a noisy, though clear, correlation between the two parameters (Fig. 3e). The variable slope of the linear regression in different preparations (color-coded dots in Fig. 3e) likely derives from the different magnitudes of spontaneous firing rates (23.0 ± 9.5 spikes/s; mean ± SD; *N* = 6 preparations) and thus on the number of isolated axons in the multi-unit recordings. While this prevents a direct correlation of absolute O_2_ levels and distinct firing rates, it confirms nonetheless that changes in neuronal activity of the central nervous system are reflected in the alterations of the O_2_ consumption.

### Alterations of O_2_ consumption during spontaneous increases of neuronal discharge

**Fig. 4.**
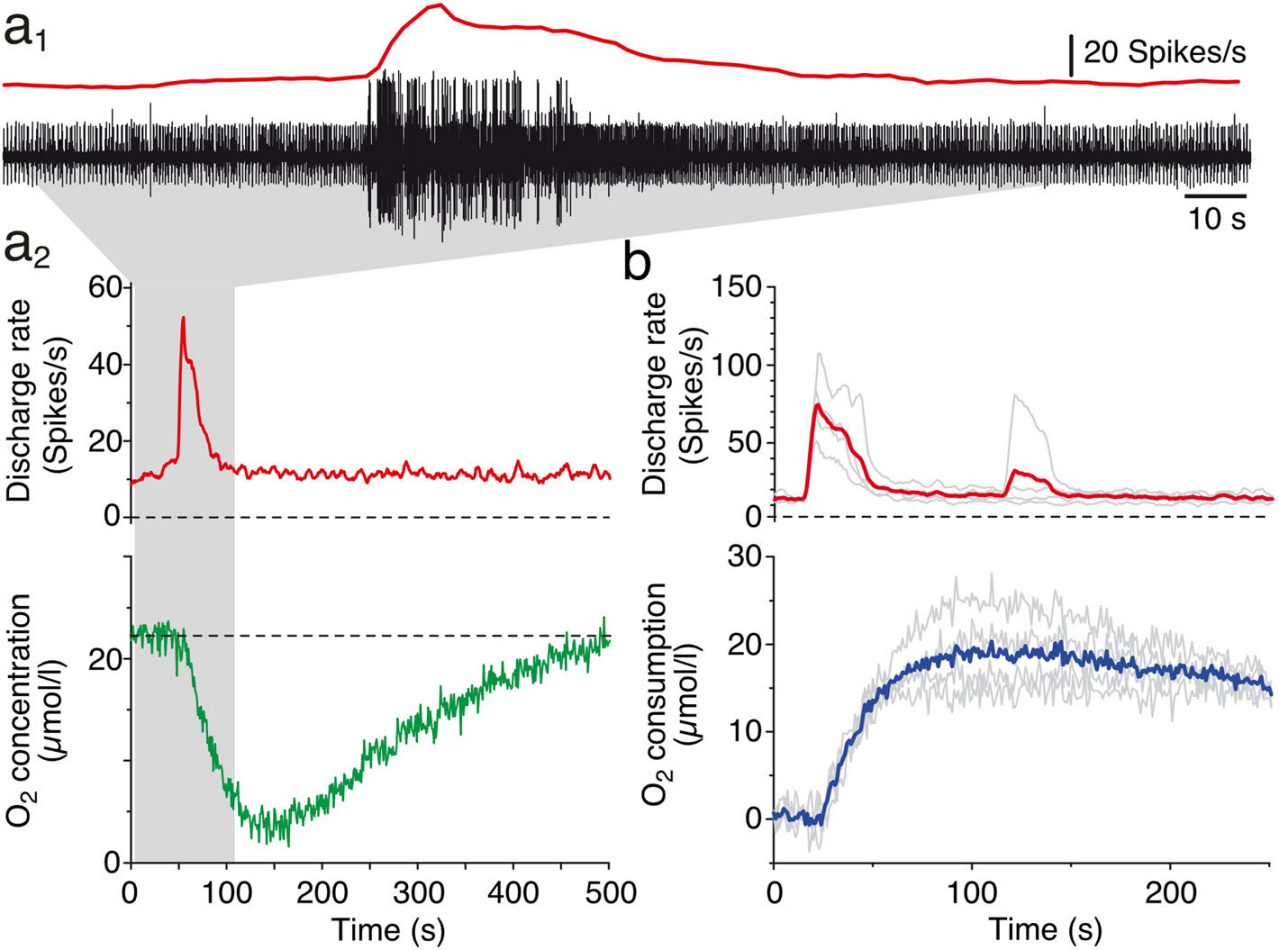
Decrease of ventricular O_2_ concentrations during spontaneous spike bursts. **a** Typical multi-unit recording of the superior oblique nerve (black trace) and corresponding firing rate (red trace; bin width 1 s), depicting a spontaneously occurring spike burst (**a**_**1**_). **a**_**2**_ shows the transiently elevated discharge rate (red trace) of the multi-unit spike activity in **a**_**1**_ (gray area) and concurrent alteration in ventricular O_2_ concentration (green trace) in an air-saturated bath solution. **b** Overlay of four consecutive spike burst episodes in an individual preparation (gray traces in the upper plot) and concurrent increases in ventricular O_2_ consumption (gray traces in the lower plot) along with the respective averages (red and blue traces)

The activity of the superior oblique nerve consists of a multi-unit discharge [37, 41] with occasionally occurring bursts of action potentials that usually last 10–60 s as depicted in Fig. 4a_1_. These spontaneous spike bursts derive from sensory-motor transformations in central vestibular neurons in the hindbrain [41] and might thus also be mirrored by alterations of ventricular O_2_ levels. In fact, augmented superior oblique nerve spike rates (red trace in Fig. 4a_1_, a_2_) are accompanied by small but robust transient drops of the residual O_2_ level in the IV^th^ ventricle (green trace in Fig. 4a_2_), indicating a burst-related temporary increase in O_2_ consumption (blue trace in Fig. 4b). Given the very low ventricular O_2_ concentrations in an air-saturated bath solution (see above), the spike burst-related increase in O_2_ consumption was consequently only in the range of 10–20 μmol/l as indicated by the averaged O_2_ transients during four consecutive spike bursts of different magnitudes in a given preparation over a period of 2 h (red and blue traces in Fig. 4b). The change in the O_2_ concentration had an average delay of ~ 15 s (14.2 ± 3.0 s; mean ± SEM; *N* = 6 preparations) with respect to the onset of the spike burst and a time to peak of ~ 100 s (98.2 ± 10.3 s; mean ± SEM; *N* = 6 preparations). The gradual return to baseline ventricular O_2_ levels usually required ~ 10 min and appeared to be correlated with the magnitude and/or duration of the spike burst. However, the generally very low baseline O_2_ levels in the ventricular compartment under air-saturated bath conditions prevented a further correlation between magnitude/temporal extent of spike bursts and O_2_ consumption dynamics. Therefore, spike burst-related alterations of ventricular O_2_ concentrations were further analyzed in preparations that were maintained at various levels of elevated bath O_2_ levels.

**Fig. 5.**
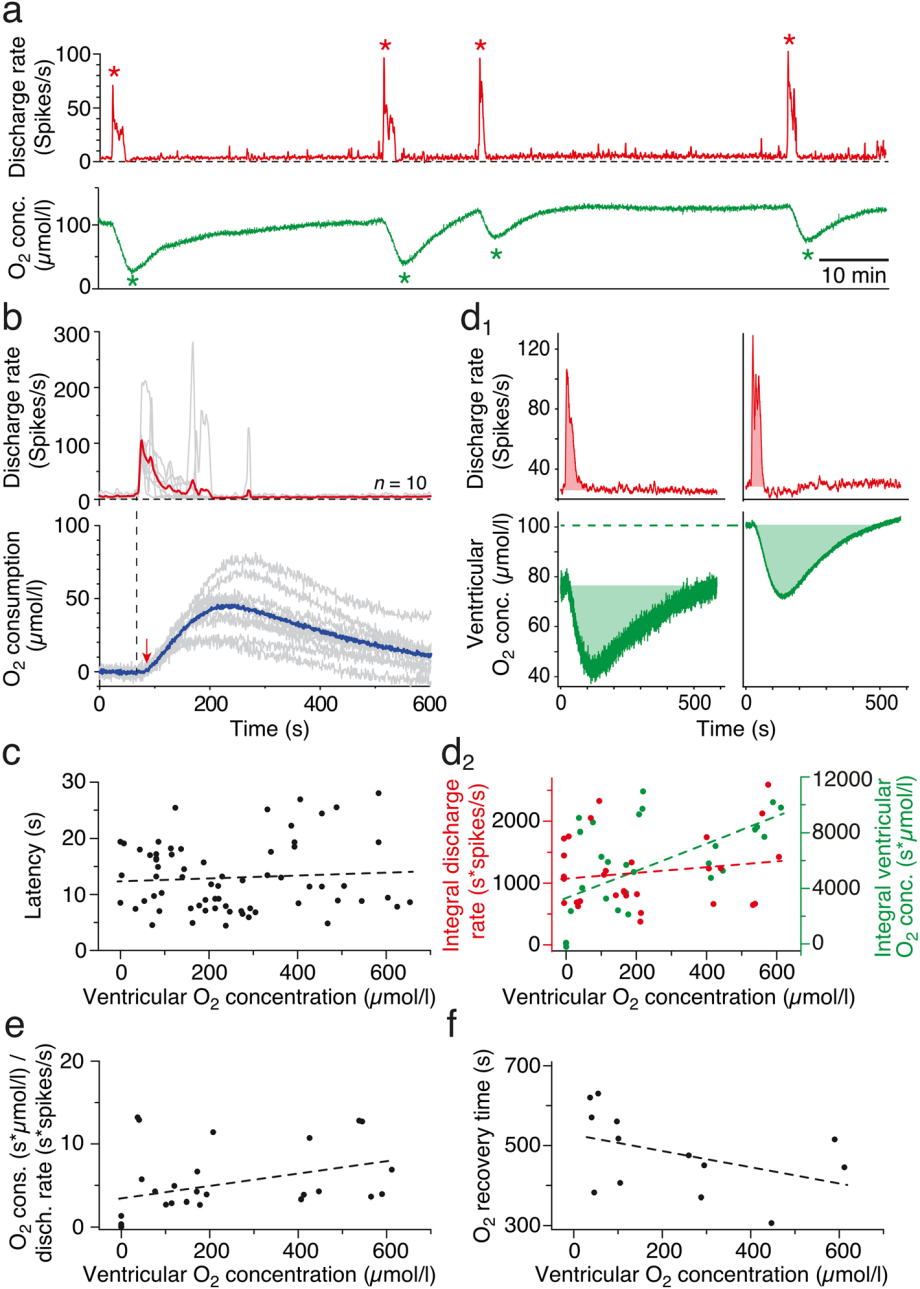
Systematic correlation between neuronal activity and ventricular O_2_ levels. **a** Superior oblique nerve firing rate profile (red trace) of four successive spike bursts (red *) and concurrent alterations of the ventricular O_2_ concentration (green trace and *) at a ventricular O_2_ level of ~ 100 μmol/l. **b** Overlay of spike burst episodes (*n* = 10) from one preparation and corresponding increases in ventricular O_2_ consumption (gray traces in the upper and lower plot) along with the respective averages (red and blue traces); vertical dashed line and red arrow indicate the latency of the O_2_ transient relative to spike burst onset. **c** Latency of O_2_ transients (*n* = 69 from 10 preparations) as function of ventricular O_2_ concentrations; the slope of the linear regression was not significantly different from zero (*r*^2^ = 0.004; *p* = 0.61). **d** Integrals of spike bursts and concurrent O_2_ transients (light red and light green areas in **d**_**1**_, respectively) at a lower (left in **d**_**1**_) and a higher ventricular O_2_ level (right in **d**_**1**_; green dashed line); **d**_**2**_ shows the dependency of spike burst (red circles) and O_2_ transient integrals (green circles) on ventricular O_2_ levels (*n* = 30 from 10 preparations); note that the integrals of the spike bursts were independent from (red dashed line; *r*^2^ = 0.03; *p* = 0.38), whereas those of the O_2_ transients significantly increased (green dashed line; *r*^2^ = 0.37; *p* < 0.001) with higher ventricular O_2_ levels. **e**, **f** Dependency of the ratio of O_2_ consumption and spike burst integral (**e**; *n* = 30 from 10 preparations) and of the O_2_ recovery time (**f**) from ventricular O_2_ levels (*n* = 13 from 6 preparations); note that the slope of the linear regression of the O_2_/firing rate (**e**) was significantly different from zero (*r*^2^ = 0.14; *p* < 0.05), whereas that of the O_2_ recovery (**f**) was not (*r*^2^ = 0.18; *p* = 0.15). *n*, number of measurements

Spontaneous episodes of spike bursts at higher bath Ringer and thus ventricular O_2_ levels were also accompanied by concurrent alterations in O_2_ concentrations (Fig. 5a), which however were considerably larger compared to those observed in air-saturated bath solutions. The overlay of ten representative spike burst episodes and concurrent O_2_ transients (gray traces in Fig. 5b) along with the respective averages in an individual preparation (red and blue traces in Fig. 5b) confirmed the impression of larger O_2_ transients with increased bath and thus ventricular O_2_ levels (~ 50 μmol/l). The latencies of the O_2_ transients after spike burst onset (dashed vertical line and arrow in Fig. 5b) at higher bath O_2_ concentrations were similar to those measured in air-saturated solutions (Fig. 5c). In fact, the average onset of the O_2_ transients of ~ 13 s (13.1 ± 0.8 s; mean ± SEM; *n* = 69 measurements in 10 preparations) was relatively independent of the O_2_ level in the ventricle (dashed line in Fig. 5c; *p* = 0.61).

A potential correlation between the magnitude of the spontaneous spike burst, associated O_2_ consumption and ventricular O_2_ level, was evaluated following calculation of the spike burst integral (light red areas in Fig. 5d_1_) and the integral of the corresponding O_2_ transient (light green areas in Fig. 5d_1_). Plotting the spike burst integral as a function of the ventricular O_2_ concentration (red circles and red dashed line in Fig. 5d_2_; *n* = 30 measurements from 10 preparations) indicated that the extent of spontaneous spike discharge was independent of the ambient O_2_ level, since the slope of the linear regression was not significantly different from zero (*p* = 0.38). In contrast, the integral of the O_2_ transient increased with higher ventricular O_2_ levels (green circles and green dashed line in Fig. 5d_2_) as indicated by the non-zero slope of the linear regression (*p* < 0.001), thus confirming the general impression of larger O_2_ transients when augmenting the bath O_2_ level (compare O_2_ transients in Figs. 4b and 5b). Normalization of the integral of the O_2_ transient with respect to the corresponding spike burst integral yielded the O_2_-consumption rate (Fig. 5e). This procedure confirmed the observed significant increase (dashed line; *p* < 0.05) of the O_2_ transient magnitude for spike burst episodes at higher ventricular O_2_ levels (*n* = 30 measurements from 10 preparations). The larger consumption at higher ventricular O_2_ levels occurred despite the tendency (the slope of the linear regression was not significantly different from zero; *p* = 0.15) of a generally faster recovery of the O_2_ transient to baseline levels (*n* = 13 measurements from 6 preparations) at more elevated ventricular O_2_ levels (Fig. 5f). Thus, the enhanced availability of O_2_ obviously dominates the increase of O_2_ consumption during spike bursts even though reacquisition of the baseline level occurs faster under these circumstances. These results represent a first demonstration of a clear correlation between spike firing magnitudes and O_2_ consumption in an isolated *Xenopus* preparation and as such the first step for further studies on more detailed aspects of the interrelation between brain activity and energy cost in such a model system (see the “Discussion” section below).

## Discussion

O_2_ concentrations in the brain of isolated preparations of *Xenopus* tadpoles were close to zero in the IV^th^ ventricle and adjacent hindbrain in air-saturated bath solutions. Application of KCN or fixation with EtOH raised ventricular O_2_ concentrations to bath levels, indicating complete consumption of available O_2_ prior to the metabolic inactivation. Artificial augmentation of bath O_2_ levels increased ventricular O_2_ concentrations, which were proportional to those in the bath above ~ 700 μmol/l. Inhibition of spike activity and spontaneous bursts caused a reversible decrease and transient increase of the O_2_ consumption, respectively.

### Suitability of isolated preparations for correlating changes in O_2_ concentration and neuronal activity

Direct in vivo measurements of O_2_ concentrations within specific compartments of the brain are usually very difficult if not impossible to obtain, mostly due to the virtual inaccessibility of the structure, the difficult targeted placement of O_2_ electrodes, the tedious maintenance of constant vital parameters of the animal, and the inability to alter O_2_ concentrations or neuronal activity in an experimentally controlled fashion. In vitro whole brain or head preparations of various vertebrate species with intact sensory organs, motor effectors, and central nervous circuits [27], which can be maintained for several days after isolation in ionically defined Ringer solutions, are highly suitable alternatives for in vivo measurements. The maintenance of isolated whole head preparations of *Xenopus laevis* tadpoles in Ringer solution [27] allows a controlled supply of O_2_ but also of other metabolically relevant molecules such as lactate or glucose through the temperature-, pH-, and O_2_-controlled surrounding bath medium. The possibility to monitor and maintain major “vital” parameters at defined levels ensures reliable and repeatable measurements under control conditions and facilitates an estimation of the consequences of experimentally altered O_2_ concentrations (Fig. 2a). The bath chamber furthermore allows an easy and fast exchange of solutions and thereby application of blockers that impair metabolic (e.g., KCN; Fig. 1f) or neuronal activity (e.g., MS-222; Fig. 3a).

The plain visibility and accessibility of the central nervous system and its ventricular compartments in isolated preparations of *Xenopus* tadpoles (Fig. 1a; e.g., [36]) allow unimpaired, μm-precise targeted placements of O_2_- and electrophysiological recording electrodes within specific brain compartments. Moreover, the vitality and functionality of isolated amphibian brains or whole heads [27] allows monitoring in vivo-like behaviors by an in vitro approach. Motor behaviors such as tail-based swimming or eye movements [42] can be evoked by close-to-natural stimuli. The knowledge of the underlying sensory-motor pathways and respective neuronal computations represents the necessary prerequisite to link patterns of neuronal activity in defined circuits with the O_2_/energy cost for respective cellular functions. The causal link between spontaneous extraocular motor spike bursts and subsequent increase in O_2_ consumption (Figs. 4 and 5) was in the current study only a first step in the attempt to calculate the energetic demand of neuronal activity for a specific motor behavior. Comparable correlations between spike activity and O_2_ tissue concentration [9, 10, 12] or turnover of energy equivalents [8, 14, 43] have been established for various neuronal populations in slice preparations. However, the latter, more reductionistic model system prevents assigning O_2_ consumption to the production of behaviorally relevant neuronal transformations or production of motor commands or behaviors. Isolated preparations of *Xenopus* tadpoles, which allow linking behavior-specific neuronal computations with concurrent alterations of O_2_ concentrations under controllable conditions, are therefore highly suitable to estimate the O_2_/energy consumption for defined neuronal activities. In a next step, superior oblique motoneurons will be activated by visuo-vestibular stimulation (sensory activation or galvanic currents) to elucidate the O_2_ dynamics during natural activation of optokinetic and vestibulo-ocular reflexes in isolated tadpole preparations. The knowledge obtained by our study now allows framing the experimental conditions such that it will be possible to visualize the O_2_ consumption dynamics during a particular motor behavior.

### Correlation between bath and ventricular/brain O_2_ concentrations

The amount of O_2_ consumed by an isolated preparation became immanent through the vertical profile of the O_2_ concentration above the head in the bath solution (Fig. 1b–d). While the O_2_ level in most of the bath volume is ~ 290 μmol/l under air-saturated conditions (red color in Fig. 1b), a hovering vertical layer of ~ 1.0 mm immediately above the brain and adjacent skull tissue reveals gradually lower O_2_ concentrations (violet in Fig. 1b) when approaching the tissue surface with the O_2_ electrode from above. The reduced O_2_ level signifies the sustained consumption by the viable tissue that is obviously faster than the resupply via diffusion from the bath, which is particularly obvious in the brain where the IV^th^ ventricle and adjacent hindbrain is virtually anoxic (blue in Fig. 1b). Accordingly, the O_2_ consumption due to the metabolic activity generates a sink with a gradient perpendicular to the tissue surface that varies in slope with the bath O_2_ level (Fig. 2b). Inactivation of the tissue with EtOH (Fig. 1d) or KCN, a potent inhibitor of the cell respiratory chain, annihilates the O_2_ sink and thus abolishes the gradient.

Independent of the bath level, the O_2_ concentration in the IV^th^ ventricle and adjacent hindbrain were identical (compare black and blue circles in Fig. 2c). Moreover, increases of bath O_2_ levels caused concurrent alterations of the respective concentration in both compartments with similar dynamics and magnitude. This indicates that neither the surface nor cellular elements form a major barrier for O_2_ diffusion into the brain. Thus, O_2_ monitoring in the ventricular compartment directly reflects the O_2_ dynamics of the adjacent hindbrain tissue and has the advantage of not damaging the neuronal tissue. The delay to reach a new steady-state ventricular O_2_ level in metabolically inactive preparations (~ 15 min; Fig. 1e) appears to be independent from the bath O_2_ level and is likely related to the consequence of the ventricular topography on O_2_ diffusion and thus likely reflects an amphibian brain-specific feature.

In air-saturated bath solutions, the O_2_ concentration in the ventricle and adjacent brain tissue was close to zero (Figs. 1 and 2). Similar low O_2_ levels were also observed in studies that employed mammalian slice preparations in air-saturated Ringer solutions [8, 26]. The virtual absence of O_2_ in isolated amphibian brains or mammalian brains in vivo [44, 45] is likely due to the efficient metabolic turnover of all available O_2_ to generate ATP. The virtual absence of O_2_ in the ventricular compartment up to bath O_2_ levels of ~ 700 μmol/l in the current study (Fig. 2c), indicates a considerable capacity of the amphibian brain tissue to use O_2_ for oxidative phosphorylation. Utilization of O_2_ by this pathway complies with the equalization of ventricular and bath O_2_ levels following the application of KCN, known to block mitochondrial activity. This identifies oxidative phosphorylation as the dominating if not exclusive O_2_-consuming process in the amphibian brain in compliance with the general importance of this metabolic pathway (e.g., [46]). The increase of the O_2_ concentration in the bath up to 1200 μmol/l without any larger alteration in the resting rate of the superior oblique nerve spike discharge excludes a potentially toxic effect of O_2_ up to this level. This allows testing the influence of O_2_ on the generation of ATP and neuronal computations over a relatively wide range.

### Correlation between O_2_ concentration and neuronal activity

The rather low tissue O_2_ levels with bath O_2_ levels of up to ~ 700 μmol/l indicates that the central nervous system, even under in vitro conditions, is obviously capable of consuming a considerable amount of O_2_, in compliance with findings in many other studies on O_2_ consumption [10, 45, 46]. While the substantial O_2_ consumption by brain tissue is well known, it is less clear, which cellular elements dominate the O_2_ turnover and how this is governed by the biophysical and morphological characteristics of the neuronal types involved. A number of studies, mostly on slice preparations, have demonstrated the necessity of adequate O_2_ levels for sustained neuronal activity and synaptic transmission [5, 12]. Other studies emphasize the need for considerable amounts of O_2_ to maintain neuron-specific resting membrane potential levels [24] and for the homeostasis of non-neuronal elements such as astrocytes [47, 48].

Bath application of the local anesthetic MS-222 in the current experiments, known to completely abolish spike discharge in fish and amphibians including isolated larval *Xenopus* preparations [36], also reduced the O_2_ consumption by ~ 50% (Fig. 3b). The most parsimonious explanation is that the homeostasis related to spike generation and repolarization at least in the isolated amphibian brain under in vitro conditions consumes about half of the available O_2_ in air-saturated bath solutions. Moreover, the recovery of the superior oblique nerve spike discharge after an abolishment of action potentials by MS-222 was linearly correlated with the O_2_ consumption in all cases (Fig. 3e). However, the apparent lack of coherence of spike rates in different superior oblique nerves with respect to O_2_ consumption (color-coded dots in Fig. 3e) is mostly due to the large variability in the number of isolated units in these multi-unit recordings. Even though the superior oblique nerve discharge is only a proxy for central nervous spike activity, these measurements clearly infer causality and allow quantitative correlations between spike rate and O_2_ consumption. The somewhat dissociated correlation between ventricular O_2_ concentration and firing rate recovery during the first phase of MS-222 washout is potentially related to the block of ion channels, although with varying sensitivity, in addition to those that contribute to the action potential generation [40, 49]. Blocking these channels might for instance affect the membrane potential and, thereby, reduce the energy and thus O_2_ consumption for the maintenance of the resting membrane potential. This therefore requires a more differentiated and extended view on the link between spike discharge, resting membrane potential, and O_2_ consumption. On the other hand, the residual consumption of ~ 50% O_2_ in the presence of MS-222 indicates that glial cell metabolism and metabolic activity principally unrelated to action potential generation in neurons utilize a similar amount of O_2_ as required for neuronal spike firing. It is well known from mammalian systems that the maintenance of the resting membrane potential, non-signaling processes, and cellular housekeeping (e.g., maintenance of cytoskeleton and membrane structure) are metabolically demanding processes [23, 24, 50]. As an example, O_2_ consumption in mouse hippocampal slices was still substantial during tetrodotoxin-blocked action potentials [10]. A further contribution to O_2_ consumption during MS-222-blocked neuronal signaling might be attributed to O_2_ diffusion into neighboring areas, where blockage of neural firing might be incomplete, which, however, was not determined in the current study.

The amount of O_2_ to sustain neuronal spike discharge at rest increased further during spontaneous spike burst activity of the superior oblique nerve (Figs. 4 and 5). The discharge of this extraocular motor nerve is an excellent proxy for correlating increased neuronal activity with the O_2_ level measured within the IV^th^ ventricle because the major presynaptic cellular generators of these spike bursts are located in the spatially adjacent hindbrain vestibular nuclei and the superior oblique motor nucleus below the floor of the IV^th^ ventricle [39]. While the transient increase in O_2_ consumption is rather small in air-saturated bath solutions, due to the very low ventricular O_2_ levels, it becomes more pronounced at higher bath O_2_ concentrations. These larger O_2_ transients, however, are not due to an increase in spike burst magnitude but likely reflect an augmentation in the fractional contribution of oxidative phosphorylation to ATP production (compared to other pathways such as glycolysis (Fig. 5d_2_). The magnitude as well as the onset of the O_2_ transient relative to the spike burst (13–15 s) is independent of the ambient O_2_ concentration. However, this delay is slower compared to respective values reported in other, although mammalian, in vitro studies [8, 9] and potentially derives from the spatial dissociation of the measurement of the burst-relevant neuronal activity in the hindbrain and of O_2_ in the ventricle.

The quantifiable correlation between spike activity and O_2_ consumption in the current study represents an ideal condition to further evaluate the link between computational capability and metabolic activity in behaviorally relevant and morpho-physiologically characterized neural circuits. Isolated preparations such as employed here provide the accessibility to such networks for physiological recordings and calcium imaging while simultaneously offering the advantage to manipulate and measure O_2_ concentrations. Therefore, this study, using a novel model system, is only a first step in the attempt to better understand how metabolic requirements and constraints affect neuronal function. Future experiments using this model system will exploit the accessibility of successive developmental stages of *Xenopus* to probe alterations in the O_2_ turnover in the hindbrain during ontogeny and further elucidate the energetic demands and costs of activating basic motor behaviors. The virtual in vivo-like experimental conditions despite isolation of the tissue and loss of vascular oxygen supply allows direct probing of the O_2_ consumption of cell populations involved in specific behaviorally relevant neuronal computations. This includes evaluation of the influence of parameters such as temperature, ionic composition of the extracellular medium, availability, and type of metabolic substrates or the relation between O_2_ availability and neuronal function. Finally, isolated *Xenopus* preparations allow exploring the capacity of photosynthetic algae [51], experimentally introduced into the brain prior to the isolation, to produce O_2_ upon illumination, which thereby supplies or even enhances the O_2_ level in the tissue.

## Conclusions

The current study reports that O_2_ concentrations can be reliably monitored over many hours in the ventricle of isolated amphibian brain/head preparations and that these values correlate well with O_2_ levels in adjacent neuronal tissue. By experimentally altering Ringer oxygenation, we directly demonstrated a saturation of the O_2_ consumption above ~ 700 μmol/l, likely due to the upper limit of O_2_-dependent energy production at this level. Given the in vivo-like experimental conditions, it is probable that a similar value would be obtained in the living animal. Bath application of a local anesthetic completely abolished spike activity and thereby revealed that half of the O_2_ consumption supplies neuronal activity. The possibility to measure at the same time O_2_ consumption and neuronal activity in an in vivo-like brain preparation under physiological conditions now allows to directly interfere with various aspects of the coupling between brain metabolism and neuronal computations.

## Methods

### Animals and experimental preparation

*Xenopus laevis* tadpoles of either sex (*n* = 81) at developmental stages 51–54 [52] were obtained from the in-house animal breeding facility at the Biocenter-Martinsried of the Ludwig-Maximilians-University Munich, Germany. Tadpoles were maintained in tanks with non-chlorinated water (18 °C) at a 12/12 light/dark cycle. All experiments were performed in vitro on isolated preparations and complied with the “Principles of animal care”, publication No. 86–23, revised 1985 of the National Institute of Health. Permission for these experiments was granted by the Regierung von Oberbayern (ROB-55.2-2532.Vet_03-17-24).

Tadpoles were anesthetized in 0.05% 3-aminobenzoic acid ethyl ester methanesulfonate (MS-222; Pharmaq Ltd., UK) in ice-cold frog Ringer solution (in mmol/l: 75 NaCl, 25 NaHCO_3_, 2 CaCl_2_, 2 KCl, 0.5 MgCl_2_, 11 glucose, and 10 HEPES, pH 7.4) and decapitated at the level of the upper spinal cord. The skin on the dorsal part of the head was partially removed, the skull opened, the forebrain disconnected, both optic nerves severed, and the choroid plexus above the IV^th^ ventricle removed [31]. The remaining central nervous system, inner ears, and eyes with extraocular muscles and respective motoneuronal innervation were functionally preserved [27]. In parts of the experiments, extraocular motor spike discharge was recorded from the trochlear nerve after disconnection from the superior oblique target muscle at the innervation site. For all experiments, isolated preparations (Fig. 1a_1_) were placed in a Sylgard-lined recording chamber that was continuously superfused with Ringer solution at a constant temperature of 17.5 ± 0.5 °C. The volume of the bath chamber was ~ 2 ml with ~ 3 mm of Ringer above the dorsal surface of the isolated preparations. The O_2_ concentration of the Ringer under air-saturated control conditions was ~ 290 μmol/l and was experimentally increased up to ~ 1200 μmol/l by ventilation with carbogen (95% O_2_, 5% CO_2_) in a separate chamber (2 ml volume) that had a rapid outflow into the recording chamber.

### Oxygen measurements, electrophysiological recordings, and pharmacology

The O_2_ concentration of the Ringer solution in the bath, in the IV^th^ ventricle, and in the brain was constantly monitored with O_2_ electrodes (Unisense A/S, Denmark) with tip diameters of 100 μm and 10 μm, respectively. Electrodes were freshly calibrated prior to each experiment using solutions with 0 μmol/l O_2_ (0.1 mol/l ascorbic acid in 0.1 mol/l NaOH), 287 μmol/l O_2_ (air-saturated Ringer solution), and 1350 μmol/l O_2_ (carbogen-saturated Ringer solution), temperature adjusted to 17.5 °C. Profiles and gradients of the O_2_ concentration were acquired by dorso-ventral depth tracks in steps of 200 μm at the rostro-caudal level of the VIII^th^ nerve at eleven medio-lateral positions, spaced by 400 μm (dots in Fig. 1a_2_, b) and symmetrically centered on the midline of the IV^th^ ventricle. The O_2_ electrode was positioned and advanced with a piezo-stepper attached to a micromanipulator (both from Sensapex, Finland).

Spontaneous multi-unit spike discharge of the superior oblique motor nerve was recorded extracellularly (EXT 10-2F; npi electronics; Tamm, Germany) with glass suction electrodes [37]. Electrodes were pulled on a P-87 Brown/Flaming electrode puller from borosilicate glass (Science Products, Hofheim, Germany), and individually broken to fit the diameter of the superior oblique nerve. O_2_ concentrations and concurrent spike activity were digitized at 120 Hz and 5 kHz, respectively (CED 1401, Cambridge Electronic Design, UK), and stored on computer for offline analysis. The neuronal activity of the isolated preparation, detected as spontaneous multi-unit spike discharge of the superior oblique motor nerve as a proxy, was blocked by bath-application of MS-222 (0.05%, 0.5%). Mitochondrial activity was inhibited by bath-application of potassium cyanide (KCN; 500 μmol/l, Merck KGaA, Germany). For control experiments on non-living tissue, isolated preparations were fixated for 12 h in 70% ethanol (Merck KGaA, Germany).

### Data analysis

Discharge rates of the superior oblique motor nerve were obtained from multi-unit spike activity using Spike2 (Cambridge Electronic Design, UK) scripts. Spike rates were obtained from the spike times in a given recording by counting all events above a pre-determined amplitude threshold. The threshold was set to ~ 1.5 × of the amplitude of the noise level and remained unchanged for a given experiment. The multi-unit resting discharge rate was determined as average frequency of the extracted events over a period of ~ 2 min. Under air-saturated O_2_ concentrations, this yielded values that ranged from 15 to 30 spikes/s in different preparations and complied with those reported earlier [37, 41]. The multi-unit spike events were plotted as discharge rates with a bin width of 1 s, from which the parameters of spontaneously occurring spike bursts such as the delay with respect to of the O_2_ concentration or burst amplitude/integral were extracted. The burst integral was determined as area under the curve following subtraction of the resting rate from the onset of the spike burst until the time when the spike burst reached again baseline level.

The O_2_ concentration in the IV^th^ ventricle or brain and the bath chamber were measured with the technical approach and sensors described above. After each experiment, the continuously monitored bath O_2_ level served to estimate the oxygen consumption by calculating the difference between the measured bath O_2_ and the concurrent ventricular/brain O_2_ concentrations. The continuous monitoring of bath O_2_ concentrations was particularly important during experiments where the Ringer was ventilated to stepwise obtain bath O_2_ levels of up to ~ 1200 μmol/l. The integral of the O_2_ consumption during spike burst activity was determined as area between the baseline and the peak O_2_ consumption starting at the onset of the change in O_2_ level and the time when the O_2_ level reached again baseline. Integrals of spike burst-related O_2_ consumption at different bath O_2_ concentrations were determined with respect to the actual bath O_2_ level during a particular experimental condition.

All above-described analysis steps were performed in Spike2 and/or in Microcal Origin 6.0G (OriginLab Corp., USA) using implemented standard algorithms for the different computations such as measurements of onset latencies or response integrals. Plotting and calculation of linear regressions was performed in Microcal Origin 6.0G; respective slopes and correlation coefficients of regression lines were indicated in the text, figures, and/or figure legends. Data are presented as dot and whisker box plots and values are indicated as mean ± SEM, if not stated otherwise. Statistical differences between experimental groups were calculated with the non-parametric Mann-Whitney *U* test (unpaired parameters; Prism, Graphpad Software, Inc., USA) and indicated as *p* values (** *p* < 0.001; *** *p* < 0.0001) in the text.

## Data Availability

The datasets used and/or analyzed during the current study are available from the corresponding author on reasonable request.

## Abbreviations

ATP: Adenosine triphosphate
EtOH: Ethanol
FAD: Flavin adenine dinucleotide
KCN: Potassium cyanide
MS-222: Tricaine methanesulfonate
NADH: Nicotinamide adenine dinucleotide
